# Preeclampsia in pregnancy and later use of antihypertensive drugs

**DOI:** 10.1007/s10654-015-0018-5

**Published:** 2015-03-18

**Authors:** Anders Engeland, Tone Bjørge, Kari Klungsøyr, Rolv Skjærven, Svetlana Skurtveit, Kari Furu

**Affiliations:** 1 Division of Epidemiology, Department of Pharmacoepidemiology, Norwegian Institute of Public Health, Oslo, Norway; 2 Department of Global Public Health and Primary Care, University of Bergen, Bergen, Norway; 3 Cancer Registry of Norway, Oslo, Norway; 4 Medical Birth Registry of Norway, Division of Epidemiology, Norwegian Institute of Public Health, Bergen, Norway; 5 Norwegian Centre for Addictive Research, University of Oslo, Oslo, Norway; 6 Division of Epidemiology, Department of Pharmacoepidemiology, Norwegian Institute of Public Health, Kalfarveien 31, 5020 Bergen, Norway

**Keywords:** Preeclampsia, Antihypertensive drugs, Prescriptions, Population-based registries

## Abstract

We explored the association between preeclampsia and later use of antihypertensive drugs in a population-based study with data from the Medical Birth Registry of Norway and the Norwegian Prescription Database. The study cohort consisted of 980,000 women having 2.1 million pregnancies during 1967–2012. Hazard ratios (HRs) with 95 % confidence intervals (95 % CI) were estimated in multivariate time-dependent Cox proportional hazards regression models. Overall, the HR of later use of antihypertensive drugs was 2.0 (95 % CI 2.0–2.0) in women with one preeclamptic pregnancy compared to women without preeclamptic pregnancies. The HR increased by increasing number of preeclamptic pregnancies, both term and preterm pregnancies. In women with two or more preeclamptic pregnancies, the HR was 2.8 (2.7–3.0). The overall HR after 40 years of follow-up for women with one preeclamptic pregnancy was 1.3 (1.2–1.4) and for two or more preeclamptic pregnancies the HR was 1.6 (1.1–2.1). The first 5 years after the first birth, the HR of being dispensed antihypertensive drugs was higher in preterm [8.4 (7.7–9.1)] than term preeclamptic pregnancies [4.3(4.0–4.6)]. However, after 10 years, this difference was no longer present. The HR of later use of antihypertensive drugs increased with the number of preeclamptic pregnancies, and in the first 10 years the HR was higher after a preterm than a term preeclamptic pregnancy. Although the HR decreased with time since first birth, the risk was still elevated after 40 years.

## Introduction

About 3 % of Norwegian pregnancies are complicated by preeclampsia (PE) [1], a condition characterized by maternal hypertension and proteinuria after the 20th week of pregnancy. The disease is a leading cause of maternal as well as foetal morbidity and mortality. Preeclampsia has in particular been shown to be associated with later maternal hypertension [2–4], ischemic heart disease, cerebrovascular disease, type 2 diabetes mellitus, chronic renal failure [5] and cardiovascular mortality [6]. Preeclampsia is one of the most deadly hypertensive complications of pregnancy and increases the risk of later cardiovascular disease two to four times [7].

Preeclampsia is associated with severe endothelial dysfunction, and these endothelial changes might increase the risk of later cardiovascular disease [8]. Williams [9] discussed how PE can predict later maternal health, and emphasized the need for women with a history of PE to make changes in lifestyle. Bellamy et al. [4], found it likely that women having recurrent PE had a pathological phenotype that puts them at risk of hypertension. After a review of 13 studies, they reported a relative risk of 3.7 of later maternal hypertension associated with a history of PE. The included studies were cohort studies (including case-cohort and nested case–control studies) with a mean follow-up time of 14 years. A population-based cohort study of 3400 women [10], it was found that women with a history of hypertensive disorders in pregnancy had higher blood pressure and body-mass index than those without. In a more recent review [11], it was reported a relative risk of 3.1 of maternal hypertension after PE.

The aim of our study was to explore possible relations between PE associated with preterm or term delivery and later maternal use of antihypertensive drugs in a large population-based cohort study with long follow-up using national registries in Norway. We also wanted to explore whether a possible relation changed with number of pregnancies with PE, number of births and time since first childbirth.

## Materials and methods

### Data sources

The Norwegian Central Population Registry (NCPR) contains demographic information on all residents in Norway, including date of birth and date of emigration or death [12].

The Norwegian Prescription Database (NorPD) [13] contains information on all prescribed drugs, dispensed at pharmacies to individual patients treated in ambulatory care, both public and private, from January 1st, 2004, and covers the entire Norwegian population (approximately 5.0 million individuals in 2012). Data on use in institutionalized patients in nursing homes and hospitals are also collected, but these figures are only registered at an institutional level. Therefore, drugs dispensed at institutions are not included in our study. For each prescription, age of the patient, demographic information, dispensing date, and detailed drug information are registered. Classification of drugs is based on the Anatomical Therapeutic Chemical (ATC) classification system [14]. In this study, we included the following drugs (hereafter denoted ‘antihypertensive drugs’): Antithrombotic agents (ATC-code: B01), antihypertensives (C02), diuretics (C03), beta blocking agents (C07), calcium channels blockers (C08) and agents acting on the renin-angiotensin system (C09). In Norway, medications for chronic diseases are reimbursed, and only reimbursed medications were included here.

The Medical Birth Registry of Norway (MBRN) is a population-based registry containing information on all births in Norway since 1967 (more than 2.7 million births), except most induced abortions. MBRN is based on compulsory notification of every birth, from 16 completed weeks of gestation onwards, and includes identification of the parents by their personal identification numbers, demographic information on the parents, maternal health prior to and during pregnancy, length of pregnancy, delivery as well as information on the infant, including birth defects and other adverse pregnancy outcomes [15]. Until late 1998, PE was notified to MBRN either as “preeclampsia” or the combination of “hypertension” and “proteinuria”. Since late 1998, MBRN defines PE as an increase in blood pressure to at least 140 systolic and/or 90 mmHg diastolic (or an increase in diastolic blood pressure ≥15 mmHg from the level measured before 20th gestational week), combined with proteinuria (at least 0.3 gram per 24 h or ≥1+ on dipstick).

From late 1998, a new notification form based on check boxes was introduced and PE was notified by the alternatives “Preeclampsia, mild”, “Preeclampsia, severe” or “Preeclampsia, before 34 weeks”, and with separate check boxes for HELLP (hemolysis, elevated liver enzymes and low platelet count) syndrome and eclampsia. The new notification form also included information on maternal smoking in association with the pregnancies, as well as ultrasound based estimation of gestational age.

In the present study, we denote pregnancies with PE (2.8 %), eclampsia (0.03 %) and HELLP syndrome (0.05 %) as preeclamptic (2.8 %). Further, PE was categorized as preterm or term PE based on whether the delivery occurred before 37 or at 37 completed weeks or more, respectively [6]. Gestational age was based on the last menstrual period (LMP) until 1998, and thereafter primarily on ultrasound estimation, but on LMP if ultrasound estimation was missing.

### Study subjects

**Fig. 1:**
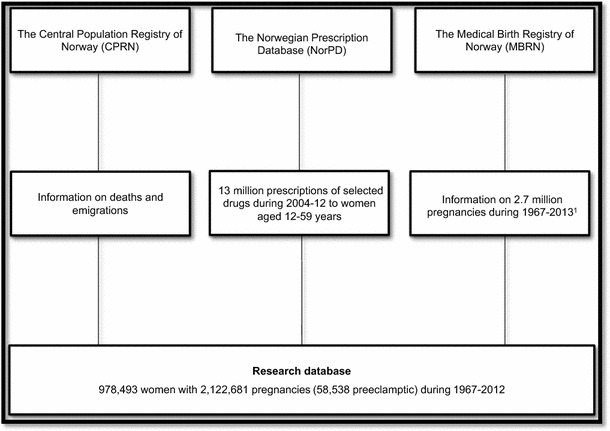
Data sources. ^1^Mothers of all singleton pregnancies ending in a birth during 1967–2012, were included in our study. However, births in 2013 were also used to have information on when new pregnancies in 2012 started, when constructing the observation time for the analyses

Data in NorPD, MBRN and NCPR were linked using the unique 11-digit personal identification number, assigned to all individuals living in Norway (Fig. 1).

In our study, mothers of all singleton pregnancies ending in a birth during 1967–2012 with birth weight of 500 g (the mean weight of foetuses at about 22 completed weeks of gestation [16]) or more, were included, provided that the mothers were still living in Norway later than July 1, 2004, and that their first birth was registered in MBRN. Births in 2013 were also used to obtain information on when new pregnancies in 2012 started for constructing the observation time until censoring.

Pregnancies where maternal heart diseases or maternal chronic hypertension were diagnosed prior to the specific pregnancy or antihypertensive drugs were dispensed to the mother prior to the specific pregnancy, were excluded (8 % of the pregnancies). Dispensed antihypertensive drugs during pregnancy were excluded as well.

In Norway, medicines for chronic diseases are usually dispensed for a maximum of 3 months at the time. To ensure that none of the women in our study population had hypertension before pregnancy we chose to exclude those having been dispensed antihypertensive drugs between January 1 and June 30, 2004. A total of 978,493 mothers remained (Fig. 1).

**Fig. 2:**
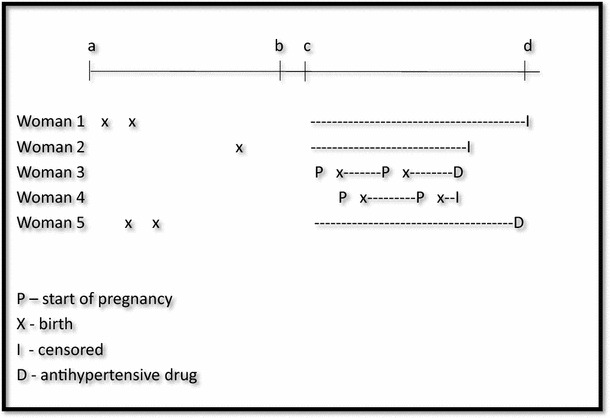
Observation time (*dashed line*) in the Norwegian Prescription Database (NorPD) for women included in the study according to pregnancies during 1967–2012. Time-dependent variables were used to update information on the women at each birth. *a* January 1, 1967, start of the Medical Birth Registry of Norway. *b* January 1, 2004, start of the Norwegian Prescription Database. *c* July 1, 2004: Earliest start of follow-up. *d* December 31, 2012, end of follow-up

Information on previous preeclamptic pregnancies was obtained from births in MBRN during 1967–2012. All mothers were followed, with respect to the dispensing of antihypertensive drugs in the NorPD, from their first registered birth or July 1, 2004, whichever occurred latest (i.e. the data were left censored), until dispensed antihypertensive drugs, emigration, death or December 31, 2012 (Fig. 2). Time during pregnancies was excluded from the observation time. In all analyses, information from all previous births was used for each woman.

### Methods

Hazard ratios (HRs) of being dispensed antihypertensive drugs were estimated using multivariate time-dependent Cox proportional hazards regression models. Time since first birth was used as time variable.

In the analyses, we adjusted for a number of potential confounders. We included previous maternal number of births, year of childbirth (1967–1976, 1977–1986, 1987–1996, 1997–2006 and 2007–2012) and number of preeclamptic pregnancies (none, one and two or more), in addition to maternal age and maternal age at first birth (<20 years, 20–24, …, 35–39 and ≥40 years). The impact of adjustment on the estimates was modest. We also made stratified/adjusted analyses including variables only available for parts of the study period (e.g. maternal smoking).

Since diabetes has been shown to be associated with the use of antihypertensive drugs, analyses with adjustment for maternal diabetes prior to pregnancy were performed.

From 1999, information on maternal smoking was available, and was used to stratify pregnancies on maternal daily smoking (yes/no).

Unfortunately, information on maternal weight and height was scarce and only available in a few years at the end of the study period. Hence, the impact of maternal weight and height could not be addressed.

The main analysis was performed both with and without antithrombotic drugs (ATC-code: B01) included in the group of antihypertensive drugs.

Data handling and analyses were performed using IBM Statistics 20 and Stata/SE 12.1.

## Results

This study included information on 2,122,681 births for 978,493 women. A total of 58,538 pregnancies were preeclamptic. Altogether 52,691 women had one or more preeclamptic pregnancies, 75 % of these women had PE in their first pregnancy. The women in the study were followed in the NorPD for an average of 6.7 years (range 0–8.5 years), constituting 6.6 million person-years. Due to the start of follow-up of women in the NorPD in July 1st, 2004, or later (since NorPD was established in 2004), the mean start of follow-up in the NorPD was 14 years after the first birth of the women (range 0–38 years), and the total follow-up was up to 46 years after the first birth. Altogether 155,978 women were dispensed antihypertensive drugs during follow-up in NorPD (Table 1). 95 % of the follow-up time was in women without any preeclamptic pregnancies (Table 2).

**Table 1:** Characteristics of the women and person-time of follow-up in the Norwegian Prescription Database (NorPD)

	Number of women	Number of women who were dispensed antihypertensive drugs during follow-up in the NorPD	Person-years	Rate of dispensed antihypertensive drugs per 1000 person-years
*Year of birth (mother)*				
<1950	116,628	42,617	787,399	54.1
1950–1959	224,187	56,234	1,649,742	34.1
1960–1969	262,100	36,635	2,042,521	17.9
1970–1979	243,403	16,752	1,611,609	10.4
1980–	132,175	3740	472,941	7.9
*Year of childbirth* ^a^				
1967–1976	97,447	35,770	656,264	54.5
1977–1986	167,829	46,711	1,211,310	38.6
1987–1996	228,088	40,670	1,756,335	23.2
1997–2006	340,147	29,171	2,565,075	11.4
2007–2012	144,982	3656	375,229	9.7
*Age* ^a^				
<20	30,045	4004	186,354	21.5
20–24	179,681	28,234	1,140,733	24.8
25–29	347,020	54,295	2,309,765	23.5
30–34	293,559	45,887	2,034,923	22.5
35–39	110,686	19,857	773,239	25.7
40–44	16,931	3567	115,593	30.9
45+	571	134	3606	37.2
Total	978,493	155,978	6,564,212	23.8

^a^At first pregnancy registered in the Medical Birth Registry of Norway

**Table 2:** Observed person-time, in the Norwegian Prescription Database, according to characteristic of the previous births^a^

	Number of women who were dispensed antihypertensive drugs during follow-up	Person-years	%	Rate of dispensed antihypertensive drugs per 1,000 person-years
*Pregnancies*				
First pregnancy	32,413	1,524,541	23	21.3
Second pregnancy	73,404	3,073,281	47	23.9
Third pregnancy	38,165	1,540,006	23	24.8
Fourth pregnancy or more	11,996	426,383	6	28.1
*Preeclampsia*				
No preeclamptic pregnancies	142,680	6,237,038	95	22.9
Preeclampsia once	11,636	295,603	5	39.4
Preeclampsia twice or more	1662	31,570	0	52.6
*Time since first birth (years)*				
0–9	14,660	1,699,935	26	8.6
10–19	27,610	1,818,055	28	15.2
20–29	41,526	1,521,496	23	27.3
30–39	58,443	1,305,013	20	44.8
40–49	13,739	219,714	3	62.5
Total	155,978	6,564,212	100	23.8

^a^A woman may be included in more than one category due to multiple births

The HR of being dispensed antihypertensive drugs increased with increasing number of preeclamptic pregnancies (Table 3). The HR was 2.0 (95 % CI 2.0–2.0) in women with one preeclamptic pregnancy compared to those without. In women with two or more preeclamptic pregnancies the HR was 2.8 (2.7–3.0). Excluding antithrombotic drugs (ATC-code: B01) from the group of antihypertensive drugs gave similar results (data not shown).

**Table 3:** Hazard ratios (HR) with 95 % CI of being dispensed antihypertensive drugs, from time-dependent Cox regression analyses

	HR	95 % CI	HR^a^	95 % CI
*Year of birth (mother)*				
<1950	1.0	Reference	1.0	Reference
1950–1959	0.8	0.8–0.8	0.9	0.9–0.9
1960–1969	0.7	0.7–0.7	0.9	0.9–0.9
1970–1979	0.6	0.6–0.6	0.9	0.9–1.0
1980–	0.5	0.5–0.6	0.9	0.9–1.0
*Parity* ^b^				
No previous pregnancies	1.0	Reference	1.0	Reference
One previous pregnancy	0.8	0.8–0.8	0.8	0.8–0.8
Two previous pregnancies	0.7	0.7–0.8	0.8	0.8–0.8
Three previous pregnancies	0.8	0.7–0.8	0.8	0.8–0.8
Four or more previous pregnancies	0.8	0.7–0.8	0.8	0.8–0.8
*Preeclampsia* ^b^				
No preeclamptic pregnancies	1.0	Reference	1.0	Reference
Preeclampsia once	2.0	1.9–2.0	2.0	2.0–2.0
Preeclampsia twice or more	2.7	2.6–2.9	2.8	2.7–3.0

^a^Adjusted for maternal age, age at first birth, year of childbirth and number of previous pregnancies with preeclampsia in addition to the variables presented in the table. Adjustments were made for each birth in each woman

^b^A woman may be included in more than one category due to multiple births

The HR was higher after preterm than term preeclamptic pregnancies (Table 4). The HR was 2.2 (2.1–2.3) in women with one pregnancy with preterm preeclampsy compared to those without preeclamptic pregnancies. When only births after 1998 were included (after introducing the new notification form in MBRN), the corresponding HR was 3.3 (3.1–3.5). When including only the time from the first birth to the next pregnancy or a maximum of 5 years, the HR after preterm PE was 8,4 (7.7–9,1) and after term PE the HR was 4.3 (4.0–4.6).

**Table 4:** Hazard ratios (HR) with 95 % CI of being dispensed antihypertensive drugs after preterm and term preeclampsia in one or two or more pregnancies, mutually adjusted, from time-dependent Cox regression analyses

	1967–2012		1999–2012	
	HR^a^	95 % CI	HR^a^	95 % CI
*Preterm preeclampsia* ^b^				
None	1.0	Reference	1.0	Reference
One	2.2	2.1–2.3	3.3	3.1–3.5
Two or more	2.8	2.4–3.3–9.8	4.3	3.5–5.2
*Term preeclampsia* ^c^				
None	1.0	Reference	1.0	Reference
One	1.9	1.9–1.9	2.5	2.4–2.6
Two or more	2.6	2.5–2.8	3.4	3.1–3.8

^a^Adjusted for maternal age, age at first birth and year of childbirth

^b^Preterm preeclampsia: preeclampsia and pregnancy length <37 completed weeks

^c^Term preeclampsia: preeclampsia and pregnancy length ≥37 completed weeks

The HR was highest in the first 5 years after the first birth and decreased gradually thereafter (Table 5). However, the women still had an increased HR 40 years after the first birth. After 40–44 years of follow-up, the overall HR of being dispensed antihypertensive drugs associated with one preeclamptic pregnancy was 1.3 (1.2–1.4), and for two or more preeclamptic pregnancies the HR was 1.6 (1.1–2.1). In the first 5 years after the first birth, the HR of being dispensed antihypertensive drugs was much higher in preterm PE [HR = 8.3 (95 % CI 7.6–9.1) and 13 (8.2–21) in one and two or more pregnancies with preterm PE, respectively] than in term PE [4.2 (3.9–4.5) and 8.1 (6.0–11), in one and two or more pregnancies with term PE, respectively]. However, after the first 10 years, there was no difference between term and preterm preeclamptic pregnancies.

**Table 5:** Hazard ratios (HR) with 95 % CI of being dispensed antihypertensive drugs after preeclamptic pregnancies in one or two or more pregnancies by time since first pregnancy, from time-dependent Cox regression analyses

Years since first pregnancy	One preeclamptic pregnancy^a^		Two or more preeclamptic pregnancies^a^	
	HR	95 % CI	HR	95 % CI
0–4	5.7	5.4–6.1	10	8.1–12
5–9	2.5	2.4–2.7	4.5	3.9–5.2
10–14	2.3	2.2–2.4	3.6	3.2–4.1
15–19	2.1	2.0–2.2	3.0	2.7–3.4
20–24	1.9	1.9–2.1	2.7	2.4–3.1
25–29	1.8	1.7–1.9	2.5	2.2–2.8
30–34	1.6	1.5–1.7	2.1	1.8–2.5
35–39	1.4	1.4–1.5	1.9	1.6–2.2
40–44	1.3	1.2–1.4	1.6	1.1–2.1

^a^Additionally adjusted for maternal age, age at first birth and year of childbirth

## Discussion

This population-based study included pregnancies registered in the MBRN during 1967–2012, with information on approximately 980,000 women with almost 59,000 preeclamptic pregnancies. An increased risk of later maternal use of antihypertensive drugs in women having had PE was observed. The increased risk decreased with time since first pregnancy, but the risk was still elevated 40 years later.

We used nationwide registries, MBRN and NorPD, based on compulsory notifications of all births in Norway and all prescribed drugs dispensed to individuals from all Norwegian pharmacies. A number of papers on the occurrence of PE have been published based on MBRN data [6, 17, 18], and the diagnosis of PE for the time period 1967–2005 was validated in 2013 [19]. The registration of PE in MBRN was found to have an average positive predictive value of 88 %, ranging from 80 to above 90 % during these years.

The exposure information (PE) was collected prior to eventual dispensing of antihypertensive drugs. Therefore, selection and recall bias were eliminated. The outcome of the study (dispensing of antihypertensive drugs) was based on NorPD using reimbursed prescriptions (71 % of the first dispensed drugs among those included were reimbursed).

Date of dispensing medications was known, but we did not know whether the dispensed drugs were actually used, or exactly when they were used. Further, we were not able to capture medications received by women during a possible hospital stay. However, women given antihypertensive drugs during hospitalization will usually be issued a prescription when leaving the hospital for continued use at least for some time. All these prescriptions will be captured when dispensed, also at hospital pharmacies. Unlike some European countries, also prescriptions issued in private health care were captured.

In this study, we used dispensed antihypertensive drugs as an indication of hypertension. To ascertain that the prescriptions were given to treat hypertension, we only used dispensed drugs that were reimbursed, indicating that the drugs were used for a chronic disease. Diabetes type II has been shown to be associated with obesity and use of antihypertensive drugs [20]. Adjustment for diabetes did only change the estimates moderately, but all types of diabetes (type I, type II, unspecified and gestational diabetes) were associated with later use of antihypertensive drugs. Another important risk factor for cardiovascular disease, smoking, reduces the risk of mild forms of PE [21]. Since 1999, maternal smoking habits have been registered in MBRN. However, adjusting for maternal smoking, or restricting the analyses to non-smokers only, did not change the association between PE and later antihypertensive drug prescriptions, although maternal risk of being dispensed antihypertensive drugs was more than 25 % higher in smoking mothers compared to non-smoking mothers in this period. These results indicate that maternal diabetes and smoking are not important when studying the relation between PE and later maternal hypertension.

We assumed that women not being dispensed antihypertensive drugs with reimbursement codes during the first 6 months of 2004, had not used these drugs previously. With this assumption, we lost women giving birth in the earlier years of MBRN who only had a transient need of antihypertensive drugs during the years before 2004.

All births with registered maternal chronic hypertension or maternal heart disease before pregnancy were excluded. Other diseases may, however, predispose to PE as well. Sensitivity analyses also excluding rheumatoid arthritis and chronic renal disease were performed, but results were similar.

In studies based on data from large population-based registries, the presence of residual confounding cannot be ruled out, because the information recorded is often grouped in broad categories. However, adjustment for the variables included influenced the results only moderately. Hence, our results should be replicated in studies including more detailed information.

The risk of later maternal hypertension increased with the number of pregnancies with both preterm and term PE in our study. In both the review by Bellamy et al. [4] and by Brown et al. [11] the studies included had shorter follow-up. In our study, the HR of hypertension associated with PE decreased consistently by time since first birth, explaining the somewhat lower estimates in our study.

Magnussen et al. [17] concluded that women with hypertensive disorders during pregnancy were more likely to develop substantially higher blood pressure several years after pregnancy, and that the risk increased with the number of preeclamptic events. In our study, we found an increased risk of being dispensed antihypertensive drugs about 40 years after preeclamptic pregnancies. We also found increased risk with number of preeclamptic pregnancies. Magnussen et al. [17] did not find substantial difference in the cardiovascular risk factor profile between women who had experienced preterm and term PE. We, however, observed that women with preterm PE experienced a higher risk of being dispensed antihypertensive drugs than women with term PE, at least during the first 10 years after the pregnancy.

Romundstad et al. [18] have suggested that the association between hypertension in pregnancy and cardiovascular risk factors could to a large extent be attributed to risk factors present before pregnancy. This might be the case, but a history of PE may nevertheless serve as a screening tool for later health problems.

In conclusion, the HR of later use of antihypertensive drugs increased with the number of preeclamptic pregnancies, and in the first 10 years the HR was higher after a preterm than a term preeclamptic pregnancy. The excess risk decreased with time since first birth, but was still increased after 40 years.

## Abbreviations

ATC: Anatomical Therapeutic Chemical
CI: Confidence interval
HR: Hazard ratio
ICD: International Classification of Diseases
MBRN: Medical Birth Registry of Norway
NorPD: Norwegian Prescription Database
PE: Preeclampsia
